# Toward a Theory of Childhood Learning Disorders, Hyperactivity, and Aggression

**DOI:** 10.5402/2012/589792

**Published:** 2012-09-27

**Authors:** Anthony R. Mawson

**Affiliations:** School of Health Sciences, College of Public Service, Jackson State University, 350 West Woodrow Wilson Drive, Room 229, Jackson, MS 39213, USA

## Abstract

Learning disorders are often associated with persistent hyperactivity and aggression and are part of a spectrum of neurodevelopmental disorders. A potential clue to understanding these linked phenomena is that physical exercise and passive forms of stimulation are calming, enhance cognitive functions and learning, and are recommended as complementary treatments for these problems. The theory is proposed that hyperactivity and aggression are intense stimulation-seeking behaviors (SSBs) driven by increased brain retinergic activity, and the stimulation thus obtained activates opposing nitrergic systems which inhibit retinergic activity, induce a state of calm, and enhance cognition and learning. In persons with cognitive deficits and associated behavioral disorders, the retinergic system may be chronically overactivated and the nitrergic system chronically underactivated due to environmental exposures occurring pre- and/or postnatally that affect retinoid metabolism or expression. For such individuals, the intensity of stimulation generated by SSB may be insufficient to activate the inhibitory nitrergic system. A multidisciplinary research program is needed to test the model and, in particular, to determine the extent to which applied physical treatments can activate the nitrergic system directly, providing the necessary level of intensity of sensory stimulation to substitute for that obtained in maladaptive and harmful ways by SSB, thereby reducing SSB and enhancing cognitive skills and performance.

## 1. Introduction

Learning disorders involve deficits in cognitive, language, and/or motor functions which adversely affect the ability to receive, understand, store, and respond to information. Resulting *learning disabilities* tend to be distinctive for specific individuals, affect a narrow range of skills and activities, and are not due primarily to mental retardation, behavioral disturbances, lack of opportunities to learn, or primary sensory deficits [1, 2]. The functions most commonly affected include the ability to read, listen, think, speak, write, spell, perform mathematical calculations, organize materials, make plans, and execute them. These skills are developed in the family but practiced and mastered in school, so that a child's self-image depends partly on success in that environment. Since teacher reports influence parents' images of their children, learning disabilities can adversely affect the quality of the child's emotional, social, and family life [3].

Learning disorders are often directly associated with hyperactivity and aggression and are part of a spectrum of neurodevelopmental disorders [4, 5]. Mild cognitive disabilities, for example, in reading, may not be associated with motor or behavioral disorders, whereas more severe learning problems tend to coexist with behavioral disorders and diagnoses that can include attention deficit hyperactivity disorder (ADHD), autism spectrum disorder (ASD), Tourette's syndrome, Fragile-X syndrome, Rett syndrome, Down syndrome, oppositional defiant disorder, conduct disorder, anxiety disorder, bipolar disorder, and antisocial personality disorder (APD). For instance, while about 30% of individuals with ASD engage in self-harming behaviors such as eye poking, skin picking, hand biting, and head banging [6, 7], persons with severe neurodevelopmental disorders have the highest overall rate of self- and other-directed violence of any diagnostic group [8]. Among longer-term prison inmates, the prevalence of adult ADHD is estimated at 40% [9].

Continuity in the association between these conditions over time is shown by the fact that at least 50% of hyperactive children develop oppositional defiant disorder, about a third develop conduct disorder before puberty, and about half of this combined group of children develop antisocial personality disorder in adulthood [10]. Among 100 adults consecutively arrested for severe crimes of violence and subjected to pretrial forensic psychiatric investigation, 55 had childhood-onset neuropsychiatric disorders (e.g., ADHD, learning disability, tics and autism spectrum disorder) as well as adult personality disorders, mood, and substance abuse disorders, suggesting that childhood-onset social and behavioral problems often precede the development of pervasive adult violent behavior [11].

Consistent with growing research interest in the continuity of behavioral development from infancy to old age, the focus of this paper is on cognitive and behavioral problems that occur in children and adults with a variety of psychiatric and neurological diagnoses rather than on the diagnostic categories themselves. Since learning disorders tend to be associated with behavioral and mood disorders, those affected are often given multiple and overlapping psychiatric diagnoses, which has led to confusion. As noted by Harris [12], it is important to make specific behaviors and cognitive patterns the focus of research rather than psychiatric symptoms lacking precise definitions and involving multiple genes. New treatments based on knowledge of the underlying neurobiology require fine-grained analyses of behavior.

Learning disabilities affect 2-3% of the general population [13] but are very common among children. In fact, rates have increased so greatly in recent decades that the U.S. is said to be experiencing a silent pandemic of mostly subclinical developmental neurotoxicity in which 1 in 88 children have an autism spectrum disorder (ASD) [14], about 1 in 10 are diagnosed with attention deficit hyperactivity disorder (ADHD) [15], and 1 in 6 children have a neurodevelopmental disorder involving learning disabilities, sensory deficits and/or developmental delays [16]. Given that functions such as paying attention, controlling one's behavior, learning, and memorizing are critical for success throughout life, these high rates of cognitive and behavioral disorder presage a lifetime of suffering for affected children and their families as well as increased future expenditures related to education, health care, and unemployment [17]. Little is known about the causes of these trends or the underlying physiological mechanisms of learning disability (http://www.nimh.nih.gov/health/publications/attention-deficit-hyperactivity-disorder/complete-index.shtml#pub8).

Treatment options for learning and associated behavioral disorders mainly include stimulant drugs, which are effective in a high proportion of cases. Assistive technologies (ATs) are also used increasingly to enhance literacy skills, but there is limited evidence for the effectiveness of these primarily computer-based technologies in young children with disabilities [18]. Meditation and especially relaxation therapies that involve bodily stimulation reportedly help to focus attention and reduce anger and aggression in people of all ages [19, 20].

## 2. Toward a Theory of Learning Disorders, Hyperactivity, and Aggression

This paper presents the theory that the co-occurrence of cognitive deficits, hyperactivity, and aggression is a neurobiological phenotype driven by increased brain retinergic (retinoid/vitamin A) activity, and that hyperactive behavior and aggression express an underlying physiological need for intense bodily stimulation. It is proposed that in, situations of acute distress, these stimulation-seeking behaviors (SSBs) are normally self-terminating via a process of sensory feedback-induced behavioral inhibition involving the activation of the mutually interactive and opposing brain nitrergic (nitric oxide) system. However, in the case of individuals with persistent learning disorders, hyperactivity, and aggression, it is postulated that the retinergic system is chronically overactivated and the nitrergic system chronically underactivated. Under these conditions the sensory stimulation generated by the subject is insufficient to activate the nitrergic system; hence, SSB continues unchecked until endogenous background increases in nitric oxide supervene. Based on this model, vigorous physical therapies providing intense but nonharmful forms of sensory stimulation, both active and passive, would be expected to activate the nitrergic system directly, thereby by-passing the process of self-induced behavioral inhibition and administering sufficient stimulation to substitute for that obtained in maladaptive or harmful ways by hyperactivity and aggression, thus calming the individual and enhancing his or her cognitive abilities and scholastic performance.

Consider some naturalistic observations on behavior that are consistent with this overall model. When humans and animals of many species are distressed or in pain, activity levels increase and intensify, sometimes to the point of causing self-inflicted injury [21, page 121]. Distressed human beings tend to be hyperactive and unable to focus on tasks that require prolonged concentration. Stress-induced activity can take many forms, ranging from binge eating and drinking to drug ingestion, hyperactivity, affiliation, and various forms of self-stimulation, depending on the species, gender, age, level of stress, and situational circumstances [22, 23], [24, pages 111–113]. For instance, in sated laboratory rats the stress of tail-pinch has diverse effects, depending on contingencies available to the animals in the testing situation [25]. Electrical stimulation of the lateral hypothalamus in laboratory rats likewise evokes a wide range of behaviors, including eating, drinking, sexual activity, gnawing, and aggression, depending on opportunities for these activities to occur, without any change in the parameters of brain stimulation [26]. The level of intensity of stress-induced behavior tends to be proportional to the severity of the arousing conditions; thus, behaviors such as overeating, drinking, or agitation occur at lower levels of distress, whereas aggression and self-mutilation occur at high levels. Stress-associated hyperactivity and aggression are usually followed by a period of behavioral quiescence and somnolence [27, 28].

Close proximity to loved ones (“social supports”) and familiar conspecifics as well as certain forms of applied stimulation have a moderating (i.e., calming) effect on individuals under conditions of threat, stress, or community disaster [29–32]. For instance, while distressed babies can be calmed by slipping an ordinary comforter into their mouth, vestibular stimulation in the form of vertical rocking movements at 60 cycles per minute is more effective, causing babies to stop crying immediately and to show obvious signs of relaxation. Bowlby [33, pages 291–295] explains this phenomenon in evolutionary-adaptive terms, suggesting that it simulates the normal walking speed of a primate mother while carrying her infant. He further notes that while the situations that elicit and terminate distress change during development, loss of attachment figures and the familiar environment continue to be among the most important causes of distress at later ages and throughout life; conversely, reunion with such figures and return to the familiar environment are the most significant stimuli in terminating distress.

Aerobic exercise similarly reduces anxiety, depression, hostility, hunger, drug-seeking behavior, heart rate, and blood pressure [34]; massage reduces hyperactivity and aggression [34], acupuncture can reduce drug use [35, 36], and electrical stimulation of low frequency and intensity, applied via needles to the conchal area of the ears (“auricular electroacupuncture”) alleviates narcotic withdrawal symptoms in human addicts [37]. Combat troops provided with opportunities for physical activity, such as search-and-destroy missions, are less likely to become psychiatric casualties than those without such opportunities, such as soldiers holed up in trenches or manning solitary observation posts. Physical action has always been considered an effective way to reduce fear [38].

These observations show that a given stressor can elicit diverse behavioral responses, while different types of stimuli can have an overall calming effect, suggesting a common denominator both in the behavioral responses to stress and the conditions that modulate it and restore a state of calm. The latter conditions include, among others, the proximity of loved ones, physical exercise, rocking, massage, and electrical stimulation.

It is suggested that the multiple behavioral manifestations of distress are stimulation-seeking behaviors (SSBs), expressing a nonspecific physiological need for intense sensory stimulation, whereas the conditions that restore or maintain a state of calm are “sensorily equivalent” in terms of providing the intensity and duration of stimulation that would have been generated by the subject's own activities. The greater the degree of perceived stress (distress), the more intense the elicited behavior becomes, suggesting that more intense stimuli are needed in order to induce a state of calm. The stimulation obtained from different behaviors is postulated to inhibit the arousal state induced by the stress. Hence, stress-induced behavior can be considered part of a negative feedback system designed to modulate the physiological arousal level induced by the stressful stimulus, a process termed behavioral inhibition by sensory feedback [39].

Although nothing can replace a missing attachment figure or love object (which at one level is a highly complex combination and pattern of different stimuli) the preceding observations suggest that alternative forms of stimulation could substitute for that obtained through stress-induced behavior and thereby prevent or reduce such behavior. The application of this concept to individuals with learning disorders associated with hyperactivity and aggression—among whom moderate-to-intense stimulation-seeking behavior is an enduring phenomenon, putatively driven by excess retinergic activity—is the subject of this paper. As discussed below, there is increasing evidence that sensory stimulation is useful in the treatment of these conditions.

## 3. Stimulation-Seeking Behavior

Intense SSB is not only manifested in response to pain, trauma, and loss, but also in response to sensory deprivation. In fact, the concept of SSB arose from reports of animals and humans undergoing prolonged social isolation and from experiments showing that sensory deprivation will induce humans and many other species to “work” actively to obtain stimulation. For instance, sensorily deprived animals press levers to be allowed to see outside their cage, and such stimulation is often preferred to conventional rewards such as food, water, and sex. Prolonged sensory restriction is also associated with profound disturbances in cognition and perception, hallucinatory phenomena, mood disturbances, hyperactivity, and aggression. If sufficiently prolonged and severe, it can cause permanent neurophysiological deficits and growth restriction [40], [24, page 94]. Case reports and animal experiments also indicate that certain forms of stimulation can correct or offset these deficits and promote development [20, 41, 42].

These studies suggest that sensory stimulation is a true physiological need, that is, essential for normal cognitive functioning and for growth and development. But the question remains unresolved of whether SSB is a separate “basic drive” in its own right, involving novelty seeking or risk taking [43, 44], or whether it is a more fundamental aspect of all motor-motivational behaviors. The author's view is that SSB is a tropism-like response, inherent in motor-motivational behavior, providing the substrate for all forms of interactional commerce with the environment. Tropisms in lower animals are involuntary orienting and directional processes that involve movement of the organism as a whole toward or away from sources of energy such as electricity, light, and magnetism [45]. Well-known botanical examples are the tendency of plant leaves and stems to bend toward light (phototropism) and the downward growth of roots in response to gravity (positive geotropism). SSB is defined by the author as any activity that enhances or facilitates contact between an organism's sensory receptors and environmental objects and surfaces; and it comprises a spectrum of intensity of movement of the body as a whole towards external sources of stimulation as well as participation by the peripheral sensory organs and receptors in facilitating the reception of sensory stimulation, for example, via pupillary dilatation, nasal flaring, and increased skin conductance. The suggestion is that there are no “basic drives” with separately identifiable mechanisms, brain loci, or behavioral manifestations. Instead, activities that are assumed to represent basic drives (e.g., eating, drinking, sex, and aggression/predation) are considered overlapping sensorial activities that form a continuum of intensity. On this view, self-directed and other forms of behavior labeled as “aggression” are intense manifestations of SSB; moreover, nutritional needs are satisfied indirectly as a result of seeking and obtaining stimulation through the senses of vision, hearing, smell, taste, and touch [24, 39, 46–49].

On traditional models of efferent organization, a child's behavioral repertoire develops in a kind of inductive process in which a basic drive such as hunger is generalized to new behaviors, based on a pairing of the new behavior with the satisfaction of a need (e.g., [50, 51]). An alternative, “deductive” type of process may be considered, analogous to Coghill's [52, 53] theory of behavioral development, whereby behavior develops in the embryo not by synthesizing local reflexes but from a “primarily integrated total pattern of action within which partial patterns arise by individuation through restriction of both the field of motor action and the field of adequate stimulation” [53]. Here the suggestion is that the “total pattern of action” starting in utero is nonspecific SSB which, over time, becomes progressively refined or “tuned” to more specialized expressions of stimulation-seeking through a process of increasing sensory differentiation in which specific tastes, sensory preferences, and cravings are derived from a general or nonspecific substratum of sensorial experience.

## 4. Aggression as Intense Stimulation-Seeking Behavior

On this theory, “aggression” can be understood as SSB above an arbitrary level of intensity, and other forms of behavior represent SSBs of lesser intensity. Actions are conventionally labeled as aggressive when it is believed by an observer that they were intended to cause harm or injury, or to destroy inanimate objects. This implies that the concept of aggression is a hypothesis formed by an observer, based on assumed injurious intent on the part of the subject. However, the hypothesis is unfalsifiable or inapplicable in almost all instances, as well as inappropriate for many rage-associated responses [24, 39, 49]. In the first place, a child's awareness that his behavior is potentially harmful only evolves some years after the first appearance of such behavior [54]. Ironically, aggressive behavior is most common among persons whose ability to formulate or verbalize intentions is limited, that is, those with cognitive impairments [55], which includes a high proportion of criminal offenders [56]. As a practical matter, it is often impossible to establish injurious intent in the mind of perpetrators at the relevant moment [57]. Memories of reasons for perpetrating violence are notoriously subject to rationalization and distortion [58], and most incidents that come before the courts are the result of impulsive actions. Fewer than five percent of all homicides are said to be both premeditated and intentional [59]. Among people who have been revived after apparent suicide attempts, self-injurious intent is often denied after the event and other reasons or motives may be cited [60].

Self-injury is common in ADHD, ASD, and in related conditions but very seldom appears to involve suicidal intent. It is believed to serve the functions of communicating a state of distress and of regulating dysphoric emotions [61]. Although self-injury is by definition a form of self-inflicted harm and may be explained by the subject as having been done for this purpose, for example, for self-punishment, such statements can be considered rationalizations for behavior that would otherwise be inexplicable to the subject himself. Self-injury is also an extreme manifestation and outcome of lesser forms of self-directed activity that may include, among others, rubbing, scratching, biting, needling, head-banging, and hair-pulling, suggesting that frank self-injury is part of a continuum of behavior in which self-inflicted injury itself is the most visible and alarming manifestation. Arguing against “rational” explanations such as a desire to hurt or punish oneself is the fact that self-injury is not limited to humans but is common among captive animals of diverse species including birds and monkeys [62], suggesting that such behaviors are responses to severe stress or sensory deprivation.

The hypothesis of aggression also fails to explain rage-associated responses directed at inanimate objects, such as banging or kicking tables and walls, stamping or rolling on the ground, smashing inanimate objects, and screaming. As with human beings, many of the behaviors shown by other animals in a state of rage have little to do with harming a prey object or opponent [63, page 248ff], [64]. Darwin [63, page 254] noted that human expressions of rage, anger, and indignation are almost universal and include pacing or jumping rapidly about, flailing the arms, shrieking and screaming at those nearby, bristling or erection of the hair, flaring of the nostrils, protruding eyes, and rolling on the ground. Gestures such as “raising of the arms, with the fists clenched, as if to strike the offender, are common …. The desire, indeed, to strike often becomes so intolerably strong, that inanimate objects are struck or dashed to the ground ….” Yet, according to Darwin, certain gestures seem “… purposeless or frantic. Young children, when in a violent rage, roll on the ground on their backs or bellies, screaming, kicking, scratching, or biting everything within reach ….” Darwin also observed that retraction of the lips and uncovering the teeth, “as if to bite the offender,” was difficult to explain, considering that the teeth are seldom used by men in fighting.

Labeling acts as aggressive assumes, more generally, an intention on the part of the subject to *do something to *an object, but in many so-called aggressive or even violent encounters the relevant flow of force may be in the opposite direction; that is, acts typically labeled as aggressive may be interpreted as expressions of a fundamental biologic response *to obtain something from *the environment, namely, to maximize the intensity of received sensory stimulation. The proposed label of stimulation-seeking behavior would apply to actions and gestures labeled as aggressive, such as slapping, punching, hitting, biting, scratching, the use of weapons, and acts such as slamming doors, hitting walls, breaking inanimate objects, stamping on the ground, shouting, and screaming. The peripheral autonomic manifestations of rage, including dilatation of the pupils and nostrils, palmar and plantar sweating, and piloerection, may serve to increase the rate and quantity of sensory stimulation.

The stimulation-seeking hypothesis predicts that the preferred targets of violence are highly stimulating. Consistent with this hypothesis, predators tend to “attack” moving, colorful, shiny, and sound-producing targets. Cats, for instance, attack moving prey objects in preference to stationary ones [65], and Siamese fighting fish (*Betta splendens*) fight and display more with responsive than with unresponsive opponents [66]. Children who cry loudly and excessively and are difficult to comfort, especially the chronically aggressive, are at increased risk of physical abuse [67], that is, of becoming the object of intense SSB [24, 68]. The stimulation-seeking hypothesis also explains the survival value of tonic immobility or “freezing.” Animals that remain motionless when confronted by predators are less likely to be attacked than those that flee, fight back, or engage in vigorous movements [69, pages 52–57]. Predators tend not to “attack” motionless and unresponsive objects, perhaps because they offer insufficient stimulation.

In summary, self-harming actions leading to injury or death may be fortuitous consequences of efforts to obtain intense sensory stimulation. This interpretation is supported by the observation that severe self-injury is often followed by expressions of calm and relief from psychological pain and a sense of having emerged from a foggy, dream-like confusional state [70].

SSB is conceived as part of a biological feedback loop in which the sensory stimulation generated by movements and other activities serves to activate brain neurotransmitter systems which inhibit those responsible for SSB. On the hypothesis of behavioral inhibition by sensory feedback, individuals of many species, when disturbed or distressed, are aroused to seek sensory stimulation through motor-motivational activities that vary in intensity and can range from increased vigilance and orienting behavior to binge-eating and drinking, drug ingestion, agitation, aggression, and self-mutilation, depending on the severity of the arousing conditions and the availability of stimulus objects. The teleological aspects of this hypothesis would not necessarily be recognizable, of course, at the level of the actual reasons or intentions of individuals in particular situations. The stimuli obtained through SSB are hypothesized to activate systems in the brain that inhibit the behavior [24, 39, 46–49], a process that may be somewhat analogous to the phenomenon of lateral inhibition in sensory systems, which is known to involve both peripheral and central aspects [71]. The greater the degree to which the underlying physiological system is aroused, the more intense (and potentially harmful) SSB must be in order to activate the system or systems that inhibit SSB.

## 5. Impact of Sensory Stimulation on Learning Disorders Associated with Hyperactivity and Aggression

Among individuals with persistent learning disorders, the associated phenomena of hyperactivity and aggression can be considered manifestations of SSB. However, instead of being a short-term response to stress or distress, intense SSB is a chronic condition due to alterations in the physiological systems that elicit and terminate the behavior. The stimulation-seeking hypothesis suggests a high degree of nonspecificity or “bio-equivalency” in expressions of SSB; for instance, it suggests that individuals with a tendency toward one type of intense SSB are prone to engage in other types. Indeed, a strong association exists between multiple forms of impulsive behavior [72, 73], often coupled with mild cognitive impairments and activities such as alcohol and illicit drug use [74], eating disorders, obesity [75], precocious or hyper-sexuality, delinquency/criminality, and injury to the self or others. These observations support the notion that learning disorders associated with hyperactivity and aggression are expressions of a generalized increase in SSB that can be considered a *behavioral syndrome* [49].

As noted, active and passive forms of sensory stimulation can induce and maintain a state of calm in conditions of acute stress. Similarly, applied forms of sensory stimulation (“environmental enrichment”) have been shown to be useful in the treatment of children with ADHD [76] and other neurodevelopmental disorders. For instance, aerobic exercise enhances overall wellbeing and self-concept [77], improves mood in incarcerated adolescents [78], and improves cognitive functions in adults with neurological disorders [79]. Exercise programs requiring prolonged and sustained exercise at a moderate level of physical exertion produce the greatest improvements in health outcomes for older adults [80]. A systematic review of the literature involving observational and interventional studies revealed a significant positive relationship over time between participation in physical activities and academic performance in children [81]. Other recent reviews have concluded that physical exercise has profound effects on brain function, leading to improved learning and memory [82], overall cognitive skills and performance in children with learning disorders [20, 83].

A recent study on the effects of a moderate- to high-intensity physical activity program showed that fitness level and motor skills, assessed by standardized tests, as well as behavior reports by parents and teachers, and level of information processing were all improved in children with ADHD after a 10-week training compared to a control period [84]. In a study to determine the effect of acute aerobic exercise on executive function, 40 children with ADHD were randomly assigned into exercise or control groups. Participants in the former group performed a moderate intensity aerobic exercise for 30 minutes whereas the control group watched a video. Neuropsychological tasks, the Stroop Test and the Wisconsin Card Sorting Test (WCST), assessed before and after each treatment, showed that acute exercise facilitated performance in the Stroop Test and improved specific WCST performances, whereas no change was seen in the control group [85].

Similarities have been noted between animal models of enriched environments and sensory integration therapy for children with neurodevelopmental disorders. It has been suggested that the essential features of the enriched environment paradigm are multiple sensory experiences, environmental novelty, and active engagement in challenging cognitive, sensory, and motor tasks [86].

The impact of regular physical exercise training on the rate of learning and blood flow to the cerebral cortex was studied in adult female cynomolgus monkeys. The training program was comparable to levels recommended for middle-aged adults and involved running on treadmills for 1 hour a day, 5 days a week for 5 months. The exercising monkeys were compared to a control group of sedentary animals and were tested for cognitive functioning using the Wisconsin General Testing Apparatus. After 5 weeks of exercise training the experimental monkeys were significantly faster than controls at mastering the cognitive skills test and had a higher level of fitness as well as a significantly increased level of vascularity in the motor cortex [87].

Over many years, Tiffany Field and her associates at the Touch Research Institute, University of Miami, have studied the effects of therapeutic massage. These studies have consistently shown that massage therapy relieves tension and anxiety, reduces hyperactivity and aggression, and increases empathic behavior in different groups of individuals that have been studied, including violent adolescents [88–92]. A study on children with ADHD that combined massage therapy and exercise similarly reported improved symptoms and recommended further research on these treatments [93]. Chiropractic therapy is also reported to improve mood, learning and behavioral impairments in children [94, 95]. Anthroposophic therapy includes special artistic and physical therapies and is also reported to improve symptoms and quality of life for children with ADHD [96].

As noted, sensory deprivation can lead to permanent neurophysiological deficits, while applied forms of stimulation can reverse such deficits and promote neural growth [41]. Earlier studies have provided evidence of considerable neuroplasticity, metabolic activation, and functional reorganization within the brain after head injury and stroke [97] and led to the notion that environmental enrichment could be used to treat multiple disorders involving developmental delays. Therapeutic massage has been found to accelerate the growth and development of preterm infants [92, 98–100]. “Sensory integration” therapies are also in wide use for children with learning and behavioral disorders [86, 101]. The earlier such treatments are administered after injury or symptom onset, the greater is the potential for improvement. For instance, patients receiving immediate physical and occupational therapy after brain injury or stroke, particularly younger patients, have improved functional outcomes compared to those whose therapy is delayed for several months [102]. Many studies have reported enhanced voluntary movement in patients with stroke following electrical stimulation. Motor recovery in some cases has been permanent [103]. Acupuncture is a form of sensory stimulation and, when combined with usual therapy, significantly enhances a broad range of functions in patients with stroke [104, 105].

## 6. Physiological Mechanisms

The discovery of the sympathetic and parasympathetic branches of the autonomic nervous system (SNS/PNS) and their associated neurotransmitters (noradrenaline/dopamine, acetylcholine/serotonin) and physiological functions was followed by the idea that short-term imbalances in the activity of these systems were associated with acute responses such as “fight or flight” and post-prandial relaxation [106, 107]. This led to the notion that long-term deficits or excesses in these neurotransmitters could account for many chronic conditions. For instance, the monoamine hypothesis proposes that low concentrations of one or more neurotransmitters in this class (i.e., dopamine, norepinephrine, or serotonin) contribute importantly to the pathophysiology of depression. Likewise, Parkinsonism is attributed to low brain dopamine and schizophrenia to increased dopaminergic activity [108].

Poor impulse control and impaired attention—the primary symptoms of ADHD—are attributed to imbalances in noradrenaline, dopamine, and serotonin [109]. The prefrontal cortex is essential for the regulation of attention, behavior, and emotion and is underactive in children with ADHD. Studies show that dopamine and noradrenaline play important roles in the regulation of the corticostriatal circuits that control high-level executive (cognitive) functions, and their functions are impaired in ADHD [110]. Psychostimulant medications, including methylphenidate and dextroamphetamine, increase brain noradrenaline and dopamine levels and are thought to improve attention, emotion, and behavior and enhance prefrontal cortical function through postsynaptic *α*(2A)-adrenoceptors and dopamine D1-receptors [111]. One suggestion is that extracellular dopamine levels are normally high in healthy individuals and yield a steady-state level of dopaminergic activity that inhibits environmental stress-induced short-term dopamine responses through autoreceptors. In ADHD, abnormally low background extracellular dopamine upregulates the autoreceptors, which tend to boost dopaminergic responses, causing children with ADHD to be hypersensitive to environmental stimuli [112]. However, the monoamine hypothesis does not satisfactorily explain how low resting heart rate is associated with violence; nor does it explain the mechanisms of stimulant drugs for treating ADHD.

Aggression is usually understood as an aspect of the “fight or flight” response to threat or danger and the result of increased adrenergic (SNS) activity. Paradoxically, however, starting early in life, children with conduct problems, and adults with antisocial personality disorder, both of which groups are prone to episodic violence, are found to have *lower* mean heart rates than controls [113]. Similar findings have been reported in children with ADHD [114, 115]. Conversely, healthy children who secrete relatively higher levels of adrenaline tend to be less aggressive, less restless, more emotionally stable, and better able to concentrate than children who secrete less adrenaline [116].

The monoamine hypothesis has guided psychiatric research and treatment for over 50 years, yet research shows that monoamine concentrations do not differ between persons with monoamine-related disease and controls, and multiple monoamines are affected in any given psychiatric condition (the “bundle damage hypothesis”); furthermore, high-affinity transporters for noradrenaline and serotonin, which ensure neurotransmitter clearance at the synapse and are the principal targets of widely used antidepressants, are ineffective due to postsynaptic neuronal damage that may be caused by environmental toxins and/or genetic predisposition [117]. However, the environmental toxins and the mechanisms that cause postsynaptic neuronal damage remain unidentified.

The efficacy of amphetamine-like drugs that increase dopaminergic activity in the treatment of ADHD and cognitive deficits has been explained on the basis that these are “low-arousal” syndromes and that both hyperactive behavior and stimulant medications serve to raise low neurotransmitter levels into the normal range. However, the origin of low heart rate (which is said to reflect low SNS arousal) and its significance for understanding persistent aggression remains obscure. These observations suggest the need for a new perspective on the data.

## 7. Retinergic and Nitrergic Systems: Master Regulators of Arousal?

While the sympathetic and parasympathetic nervous systems and their associated neurotransmitters control short-term autonomic functioning, there is growing evidence for the existence of a more fundamental physiological regulatory system underlying metabolism, mood, cognition, and behavior. It is proposed that this system involves interactions between endogenous retinoids and nitric oxide.

Retinoids are natural and synthetic compounds related to vitamin A and its major metabolite, retinoic acid (RA). Retinoids are mainly dietary-derived, fat-soluble signaling molecules that are essential for normal cellular homeostasis, embryonic development, tissue differentiation, growth, and mucus secretion [118]. Reviews of the literature [119, 120] indicate that retinoids are important modulators of neurogenesis as well as neuronal survival and plasticity in postembryonic and adult brain; both retinoid deficiency and excess result in neuronal defects; retinoids are also important in learning and memory.

Vitamin A is derived from animal and plant sources in the diet, stored primarily (80%) in the liver, and transported to the target tissues by retinol-binding protein (RBP). The production of RA from retinol involves the following steps. Retinol is first oxidized to retinaldehyde via an alcohol dehydrogenase; secondly, RA is synthesized from retinaldehyde primarily within the cell microsomes via the enzyme retinaldehyde dehydrogenase. Serum retinol levels remain stable due to a transport system in the liver that maintains needed concentrations in the target tissues despite major fluctuations in dietary intake [121]. Retinoids act through interaction with two basic types of nuclear receptors: the retinoic acid receptors (RARalpha, RARbeta, and RARgamma) and retinoid X receptors (RXRalpha, RXRbeta, and RXRgamma), which work with other proteins to regulate the expression of specific target genes and thereby control development, homeostasis, and metabolism [122].

Nitric oxide (NO), also known as endothelium-derived relaxing factor (EDRF), is a gas that occurs in biological systems, crosses cell membranes and plays a role as a neurotransmitter in the brain. It is involved in learning processes and memory formation and in a variety of other neural functions, including cortical arousal, nociception, food intake, penile erection, yawning, blood vessel dilatation and immunoregulation [123]. Neurons synthesize NO in response to the activation of N-methyl-D-aspartate (NMDA) receptors by the amino acid glutamate. NO is generated in neurons as a product of the conversion of the precursor amino acid L-arginine to L-citrulline by the enzyme nitric oxide synthase (NOS). Distinct forms of NOS are found in endothelial cells (eNOS) and neurons (nNOS). The action of NO is not due to binding to membrane-associated receptors but diffuses from neuron to neuron, acting directly on intracellular components. NO functions as a neurotransmitter by stimulating soluble guanylyl cyclase to form the second messenger molecule, cyclic guanosine monophosphate (cGMP), in the target cells. Studies on the effects of agents that increase or decrease NO concentration in specific brain regions indicate that NO plays a major role in learning and memory [124], activating “the computational ability of the brain” [125, page 476].

Like retinoic acid, NO is a two-edged sword in that physiological levels are required for normal functioning whereas excess NO produces neurotoxicity. Inhibition of NO decreases acetylcholine release and impairs learning, indicating that cholinergic activity is involved in the cognitive effects of NO. NO production is impaired by stress and in pathophysiological conditions, including chronic renal failure, essential hypertension, diabetes, and epilepsy and appears to affect cognitive function in these conditions. NO production is also decreased in aging and in Alzheimer's disease. In sum, the nitrergic system corresponds to nitric oxide (NO) produced from neuronal nitric oxide synthase (nNOS) and functions as a brain neurotransmitter, with roles in nonsynaptic interneuronal communication as well as excitotoxic neuronal injury at higher concentrations [125].

There is evidence that the retinergic and nitrergic systems have a reciprocal inhibitory relationship, in that retinoic acid (RA) can inhibit NO [126, 127] and vice versa [128]. On the present hypothesis of inhibition by sensory feedback, retinergic activity induces progressive increases in SSB and cognitive dysfunction, and the sensory stimulation generated by SSB leads to retinergic inhibition (and to reduced SSB and improved cognition) via the activation of brain nitrergic systems. Nitric oxide synthesized from the amino acid L-arginine by nitric oxide synthase is linked to vasodilation and to improved learning and memory in experimental animals; conversely, impaired cognition due to pathological conditions such as epilepsy, stress, diabetes, and the side-effects of drugs is associated with defective NO activity in the brain. These effects can be reversed by L-arginine and NO donors. There are also reports that cognitive impairments associated with aging and with Alzheimer's disease, due to deposition of the toxic protein, beta-amyloid, can be improved by L-arginine and NO donors [125]. The NOS1 gene encoding NOS is a candidate gene for ADHD, a highly heritable disorder, and has been linked with impulsivity and reduced ventral striatal activity [129].

Retinoids interact with the classical monoamine neurotransmitters. For instance, the dopaminergic system is regulated by retinoic acid in the embryo but the influence of RA on dopaminergic systems in the adult brain is less well understood. RA appears to decrease noradrenaline in the short term by inducing the noradrenergic receptor which terminates noradrenergic neurotransmission through reuptake of the neurotransmitter [130]. RA also interacts with the human serotonin transporter gene [131]. In summary, retinoids and NO operate inversely; they are closely related to the classical, faster-acting SNS, and PNS and associated neurotransmitters and may have greater relevance for understanding chronic diseases and behavioral disorders than the adrenergic and cholinergic systems.

## 8. Implications for ADHD and Associated Conditions

**Figure 1:**
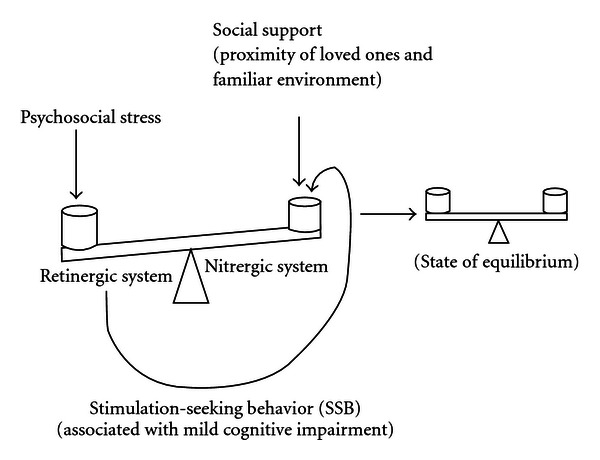
Hypothesis of inhibition by sensory feedback, involving the interaction and balance-type relationship between retinergic (retinoids/vitamin A) and nitrergic systems (nitric oxide/NO).

Stress-induced alterations in the postulated balance between retinergic and nitrergic systems are assumed to be short-lived and modulated to a considerable extent by the proximity of loved ones, the familiar environment, and other forms of stimulation (see Figure 1).

Responses to short-term psychosocial stress are hypothesized to involve (1) an initial increase in retinergic activation, which increases the intensity of stimulation-seeking behavior (SSB) and induces mild cognitive impairment. (2) The sensory stimulation thus generated activates the opposing nitrergic system, which inhibits the retinergic system, reduces SSB, leads to a state of calm, and improves cognitive functions. (3) The presence of familiar stimulus objects (“social supports”) modulates the retinergic system response to stress by activating the nitrergic system, which inhibits retinergic activity and reduces the intensity of SSB.

**Figure 2:**
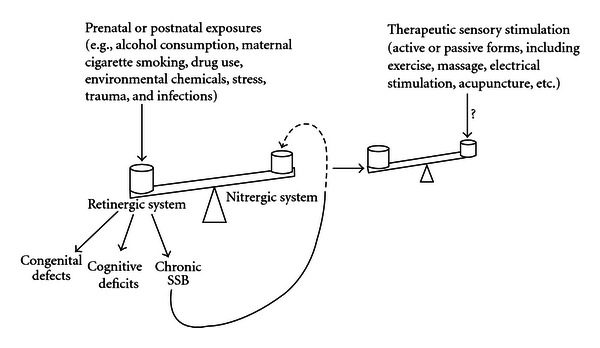
Proposed model of the pathogenesis of learning disorders associated with hyperactivity and aggression.

These generalizations may also apply to persons with ADHD, ASD, conduct disorder, and related conditions. However, it is proposed that in these conditions short-term alterations in SNS/PNS activity occur against a background state of chronic retinergic overactivation, coupled with a chronic state of nitrergic underactivation. While this hypothesis awaits direct study, it suggests that the sensory stimulation generated by SSB is insufficient to activate the nitrergic “brake” over the retinergic system, with the result that SSB continues unabated at a high level of intensity (indicated by broken line in Figure 2, see below).

It is suggested (as indicated by the question mark in Figure 2) that therapeutic sensory stimulation would reduce chronic SSB (i.e., hyperactivity and aggression) and improve cognitive functioning by directly activating the nitrergic system.

On this hypothesis, the efficacy of stimulant drugs in the treatment of ADHD and related conditions may be due to the activation of nitrergic systems and the resulting inhibition of retinoid activity and SSB. In fact, there is evidence that amphetamines increase brain nitrergic activity [132]. The dopamine-dependent psychomotor and other effects of amphetamines are also reduced by pharmacological blockade of nNOS or deletion of the nNOS gene, supporting the role of NO in synaptic plasticity and its function as a neuronal messenger [133]. The psychomotor effects of amphetamine and methylphenidate (MPD/Ritalin) are thought to depend on increased extracellular levels of dopamine (DA) in mesocorticolimbic and mesostriatal pathways. However, given the observations that (a) retinoids affect dopaminergic activity, (b) drugs that facilitate dopaminergic transmission induce cognitive and attentional deficits [130], and (c) NO modulates the effects of dopamine [134], the efficacy of stimulants may be due to NO-induced inhibition of retinoid activity, which in turn inhibits dopaminergic activity. The effects of RA are not always immediate and may require several weeks to occur, suggesting that they are due to a secondary phenomenon, perhaps involving accumulation over time. Such effects may involve initial induction of the dopaminergic system resulting in negative feedback and a long-term decline in some elements of dopaminergic transmission [130, 135], which may have implications for conditions associated with loss of brain dopamine.

The suggestion that retinergic overactivation controls SSB implies an arousal state very different from that of SNS/adrenergic arousal. Instead of “fight or fight” caused by adrenergic arousal, reports of hypervitaminosis A—unwittingly induced in early Arctic explorers by consuming vitamin A-rich polar bear or seal liver—included symptoms of drowsiness, irritability, severe headache, nausea, and various forms of “irrational” behavior. This condition was familiar to the Eskimos and was called *pibloktoq* [136]. The somatic and behavioral manifestations of hypervitaminosis A are said to have a close parallel both in the symptoms of Western patients diagnosed with hysteria and Inuit sufferers of *pibloktoq*. Eskimo nutrition provided abundant sources of vitamin A through the ingestion of livers, kidneys, and fat of arctic fish and mammals, where the vitamin is stored in poisonous quantities [137].

Unlike SNS arousal, in which all of the senses are heightened, analytical faculties are at their sharpest, and the skeletal muscles are primed for strong and decisive action, the retinergic arousal state is akin to one of intoxication. Recalling the behavior of animals subjected to tail-pinch or electrical stimulation of the lateral hypothalamus, the subject may voraciously consume alcohol or preferred foods and become hyperactive, impulsive, and highly distractible. Less commonly, the subject may self-mutilate or frenziedly attack other people.

Reports of criminal violence indicate surprising similarities to those induced by hypervitaminosis A. Many such accounts describe violent acts as being carried out in a state of diffuse cognitive impairment, perceptual distortion and anhedonia, and in a semisomnambulistic, dream-like state from which the subject later emerges, often with little or no memory of the acts or events in question [138], [24, pages 127–154]. Criminal acts associated with altered states of consciousness are occasionally reported in persons with diabetes, a condition also associated with altered retinoid metabolism [139]. Incarcerated offenders with antisocial personality disorder are reported to have abnormalities in brain glucose metabolism, indicated by decreased glucose uptake in the prefrontal cortex and a low blood glucose nadir in the glucose tolerance test [140]. These observations suggest that ADHD, ASD, conduct disorder, and related conditions are part of a spectrum of neurodevelopmental disorders reflecting chronic states of subclinical retinoid neurotoxicity due to pre- and/or postnatal exposure to excessive retinoid concentrations or expression, as discussed below.

## 9. Neurotoxicity due to Early Exposure to Excess Retinoid: Cause of the ADHD Phenotype?

Retinoids are essential for the maintenance of the placenta and for normal embryogenesis and therefore play a critically important role in maternal-fetal physiology [141–143]. Retinoic acid, the major vitamin A derivative, regulates the transcription of about one-sixth of the human genome. There is strong evidence that RA plays a role in cognitive activities, but its involvement in the molecular mechanisms of higher brain functions is not well known. In the functional cerebral cortex, RA signaling affects the expression and regulation of hundreds of genes [144] through the retinoic acid receptor (RAR) and retinoid X receptor (RXR) classes of ligand-dependent transcription factors, thereby influencing the pattern formation of many organs and tissues [145].

Low concentrations of vitamin A are essential for multiple biological functions, including cognitive and behavioral development. It has been proposed that behavioral development originates as global or nonspecific SSB and differentiates into multiple manifestations of motivational behavior with progressive maturation and sensorial experience. On the other hand, moderate-to-high concentrations of vitamin A can be prooxidant, cytotoxic, mutagenic, and teratogenic. Vitamin A toxicity is generally associated with increased levels of retinyl esters (the storage form of the vitamin) circulating with plasma lipoproteins unbound to retinol-binding protein (RBP). Retinyl esters react more randomly with cell membranes than the physiologically sequestered RBP and hence are a major form of vitamin A toxicity. Fasting retinyl ester concentrations >10% of total circulating vitamin A (retinol plus esters) are considered a biomarker for toxicity [146, 147].

Environmental exposures leading to alterations in physiological concentrations of retinoids are associated with birth defects and fetal loss [148]. There is also evidence that high serum concentrations of retinoids resulting from dietary intake, vitamin A supplements, and therapeutic retinoids are causally associated with cognitive impairments, mood disorders (e.g., depression), persistent agitation, suicide, and other forms of violence. This evidence includes case reports, proof of a temporal association between exposure and onset, challenge and rechallenge cases, similar effects resulting from different compounds in the same class of drugs, dose-response associations, and biological plausibility [130, 149, 150]. In animal models, the retinoic acid isomer 13-cis-RA induces depression-related behavior and impairments in spatial learning and memory [131]. Earlier studies provided evidence of permanent learning disability in otherwise normal rat pups whose dams received nonteratogenic doses of vitamin A (40% of the dose that causes terata); the same was true of children whose mothers received large doses of 13-cis-retinoic acid during pregnancy [151].

Regarding the mechanism of retinoid-associated cognitive impairment (i.e., irritability, anxiety, and depression), de Oliveira et al. [152] studied the effects of vitamin A supplementation at clinical doses on rat hippocampal function and found evidence of mitochondrial impairment, decreased brain-derived neurotrophic factor levels and dopamine D2 receptor levels, and decreased glutamate uptake in the vitamin A-treated rats.

An animal model has been developed that has particular relevance to understanding ADHD, ASD, and related conditions, supporting the concept that early exposure to excess retinoic acid can induce a behavioral syndrome of agitation, distractibility, and cognitive deficits. These adverse effects on fetal development and behavior resulted from exogenous retinoid concentrations that were only slightly above the normal physiological range. RA in these doses caused lasting behavioral abnormalities that suggested disruption of limbic functions. These abnormalities included extreme aggressiveness, hyperactivity, stereotypy, persistent and obsessive behavior, increased motor responses (sensitivity) to touch, sound or other disruptions, and poor grooming. The first and most obvious symptom was rapid running in circles. The movements were well-coordinated except for stereotyped head-bobbing. Some animals continued these behaviors throughout the period of observation. Many males also showed signs of mild motor weakness. The hyperactive mice could breed but failed to touch the pups and showed a lack of grooming activity. These observations suggested that the early postnatal mouse brain is highly sensitive to retinoic acid. Histological findings were dose-dependent and included signs of impaired lung function and brain swelling, most markedly in the hippocampus [153].

It is recalled that the “low arousal” hypothesis sought to explain the association between low heart rate and aggression in antisocial personality and conduct disorder by suggesting that hyperactivity/aggression were responses designed to increase heart rate. However, the origin of low heart rate itself remained unknown. An alternative hypothesis has been suggested by the author: namely, that low resting heart rate and chronic aggression are both due to prenatal exposure to excess retinoids [154]. As noted, retinoids play important roles in embryogenesis, including neural and cardiovascular development [155], and in disorders of cognition, mood, and behavior. It is reported that retinoid overexpression in utero can induce alterations in cardiac functioning and hemodynamics that include lowered heart rate [156]. Raine [113] suggested that prenatal exposure to nicotine induces low resting heart rate due to a neurotransmitter “switch” resulting from disruption of brain noradrenergic (NA) systems and enhancement of cholinergic (ACh) functioning. There is evidence that this effect of nicotine is mediated by retinoids. For instance, in NB69 neuroblastoma cells RA treatment induces a noradrenergic-to-cholinergic switch whereby tyrosine hydroxylase activity and noradrenaline levels are reduced, the expression of choline acetyltransferase is simultaneously enhanced, and the cells acquire the ability to release [(3)H] acetylcholine in a calcium-dependent fashion [157]. The induction of a cholinergic phenotype in cells treated with RA is also accompanied by a decrease in several catecholaminergic characteristics such as tyrosine hydroxylase and protein expression and activity, supporting the existence of a switch controlling the adrenergic-cholinergic phenotype [158]. Retinoid overexpression in utero could thus be responsible for alterations in cardiac functioning and hemodynamics, including low heart rate, as well as brain structural and functional changes, cognitive impairments, minor physical anomalies, and chronic hyperactivity and aggression seen in conduct disorder and antisocial personality disorder.

Alterations in retinoid metabolism and retinoid overexpression can result not only from vitamin A intake, vitamin A supplements, and treatment with vitamin A derivatives, but also from exposure to a wide range of factors, such as drugs, alcohol, cigarette smoking, environmental chemicals, and infections. Retinoid toxicity occurring early in pregnancy could thus represent a final common pathway by which numerous prenatal challenges result in a spectrum of neurodevelopmental and metabolic disorders—exposures such as prenatal stress, which is known to increase the risk of later psychopathology such as ADHD, conduct disorder, aggression or anxiety [159], and prenatal exposure to alcohol and nicotine, which is similarly associated with later conduct disorder and violent offending [113].

Alcohol consumption could induce retinoid toxicity and violence due to interactions with the retinoid system via effects on the liver and the transport of retinoids released from hepatic storage to the brain. Consistent with this hypothesis, acute exposure to 6.5% ethanol was reported to increase endogenous RA concentrations in the liver, testis, serum, and particularly the hippocampus of pregnant mice; blood alcohol concentrations also correlated with increases in RA, suggesting that increased retinoid concentrations contribute to ethanol-induced disorders, including fetal alcohol syndrome and adult cognitive disorders [160].

Smoking during pregnancy is associated with a two- to over fourfold increased risk of later conduct disorder [161], a more than twofold increased risk of ADHD [162], and increased risks of low birth weight, reduced lung function, congenital malformations, sudden infant death syndrome, cognitive impairment and mood disorders in children [163]. There is also considerable evidence that low birth weight—especially preterm birth and small-for-gestational-age birth, indicating fetal growth restriction—is an independent risk factor for motor and cognitive impairments in childhood [164]. Subtle but detectable neurocognitive deficits involving working memory and response inhibition are also reported in late preterm children [165].

Studies on the mechanisms of maternal smoking-associated later-onset diseases have focused on nicotine, which is believed to cause these effects in the offspring partly by disrupting vitamin A metabolism and lowering the availability of retinol [166]. Nicotine also disrupts the development of the noradrenergic neurotransmitter system [167] and interferes with neuronal development in the cerebellum, which in turn may affect cognitive functioning. An alternative hypothesis suggested by the present theory is that cigarette smoking during pregnancy results in neurocognitive and other adverse outcomes due to maternal liver damage and the spillage of stored retinoid compounds into the fetal circulation. This hypothesis is supported by evidence that habitual cigarette smoking is associated with hepatic inflammation, fibrosis, and increased liver enzyme concentrations [168]. Smoking-associated hepatic damage was also reported by Li et al. [169], who noted that circulating levels of retinol were reduced. It was inferred by these authors that lung damage, the subject of their study, was due to vitamin A deficiency. However, retinol concentrations can be low as a result of liver damage and impaired hepatic mobilization of the vitamin, whereas other retinoid compounds, which are seldom measured, may be found in high concentration due to spillage from the damaged liver. As noted, retinoid toxicity can occur when circulating retinyl esters, unbound to protein, exceed the carrying capacity of RBP, and an accepted indicator of toxicity is percent retinyl esters >10% [147]. On the present theory, while smoking in pregnancy is associated with reduced retinol concentrations due to liver damage, normally stored but potentially toxic retinyl esters and retinoic acids are spilled into the circulation in bile, causing growth restriction and well as later-appearing neurocognitive deficits, hyperactivity, and aggression. Support for this concept comes from a study of retinoid profiles in patients with cirrhosis [170]. Serum retinol concentration was low, but the percentage of serum retinyl esters as a fraction of total vitamin (retinol plus esters) was 20%, double that of the accepted indicator of vitamin A toxicity (>10%).

In an animal study by the author and colleagues [171], designed to test the hypothesis that gestational diabetes, preeclampsia, and fetal growth restriction are associated with maternal liver damage and retinoid toxicity, the drug streptozotocin was administered to rats early in pregnancy. Pup weights were significantly reduced, indicating fetal growth restriction. Liver damage, indicated by elevated liver enzymes, was accompanied by a significantly lower median plasma retinol (ROL) concentration, consistent with earlier studies. However, it was noted for the first time that the retinyl ester-to-ROL ratio, the percentage of retinyl ester to total vitamin A (median, 24% versus 11%; *P* = 0.008), and the concentration of RA in the experimental animals were all significantly higher compared to those of the controls. These findings support the concept that fetal growth restriction associated with cognitive deficits and behavioral disorders in later life could be due to exposure to endogenous retinoids in utero and to the growth inhibitory and other toxic effects of these compounds. 

Other factors can affect retinoid metabolism in early pregnancy in addition to alcohol and nicotine, including many medications, chemicals, illicit drugs, stress, trauma, and infection. Environmental chemicals can affect embryogenesis, immunity, and epithelial functions and interfere with retinoid metabolism and signaling. Some of the toxic effects of pesticides, polychlorinated dioxins, polychlorinated biphenyls, polycyclic aromatic compounds, and other organic pollutants may involve interactions with retinoid metabolism, transport, or signal transduction [172].

Certain neurodevelopmental disorders, in particular ASD, may not necessarily be associated causally with exposures occurring exclusively in utero. In a proportion of cases, these conditions could be triggered by exposures occurring postnatally, possibly against a background of susceptibility due to genetic influences or events during pregnancy. Challenges during early development can lead to permanent neurodevelopmental and immunological abnormalities. For instance, aluminum is an established neurotoxin, and aluminum-based adjuvants are extensively used as immune stimulants in vaccines. However, the toxicology and pharmacokinetics of aluminum adjuvants in vaccines are unknown [173]. In fact, much remains to be learned about the impact of vaccination on children's health and neurological development, a subject of great controversy and public interest because children are required to be vaccinated in order to attend daycare and school and because vaccination is assumed to be safe and effective. Many epidemiologic studies have shown no association between measles, mumps and rubella (MMR) vaccination and autism [174]. There is, however, considerable evidence that vaccines can cause autism and related neurodevelopmental conditions in certain individuals [175]. Male infants receiving hepatitis B vaccine during the first month of life were reported to have a threefold increased risk of neurodevelopmental disorders compared with those vaccinated later or not vaccinated [176]. Multiple vaccinations can precipitate developmental regression in susceptible individuals [177], and routine vaccination has been associated with a variety of autoimmune conditions [178, 179]. Urgent remaining questions are (1) the extent, if any, to which vaccination may contribute to a spectrum of brain damage and other chronic illness in children and (2) the interactive effect of different vaccines and nutritional supplements on children, in particular, vitamin A, which can reduce infant mortality associated with measles vaccine given at 9 months of age but increase it in association with diphtheria-tetanus-pertussis vaccine given between ages 1 and 5 months [180].

A central hypothesis of this paper is that the “fetal origins of disease” [181, 182] are importantly related to maternal liver dysfunction in pregnancy. On this hypothesis, liver damage-induced alterations in retinoid metabolism and exposure serve to “program” the fetus to develop later-onset conditions and diseases, depending on the timing and duration of exposure and on the concentration of circulating retinoid compounds (retinyl esters and retinoic acids). The resulting spectrum of adverse birth outcomes could range from stillbirth and birth defects to preterm birth and fetal growth restriction and their sequelae. For instance, among infants born preterm, small-for-gestational age or with minor physical anomalies, later-onset neurodevelopmental, and metabolic disorders may be long-term outcomes of exposures occurring in utero. However, in a proportion of cases, for example, children developing autism spectrum disorders after a period of apparently normal growth, postnatal liver damage-induced alterations in retinoid metabolism may trigger brain and/or other organ (e.g., intestinal) damage, possibly against a background of increased susceptibility to encephalopathy due to exposures occurring in utero. These hypotheses on the pathogenesis of neurodevelopmental disorders could be tested prospectively, for example, in the ongoing National Children's Study, which is examining the effects of the environment, cultural influences and genetics on the growth, development, and health of children across the United States, following them from before birth until age 21 years (http://www.nationalchildrensstudy.gov/Pages/default.aspx).

## 10. Self-Injury: The Case of Lesch-Nyhan Syndrome

Self-injurious behavior (SIB) occurs in several neurodevelopmental and psychiatric disorders, including autism, conduct disorder and bipolar disorder as well as in neurogenetic syndromes such as the Lesch-Nyhan, Prader-Willi, Rett, Cornelia de Lange and Tourette syndromes, familial dysautonomia, choreoacanthocytosis, sensory neuropathy, and nonspecific mental retardation. SIB includes acts such as head banging, eye gouging, finger or lip biting and self-hitting, hair pulling, and skin picking. In some of these conditions the manifestations are relatively specific; for example, stereotypical skin picking accompanies the hyperphagia of Prader-Willi syndrome, recurrent finger and lip biting are typical of Lesch-Nyhan disease, and head banging is common in children with autism. The biological basis of SIB is obscure, and present treatments are unsatisfactory [183].

The retinergic-nitrergic model suggests that SIB is associated causally with alterations in the metabolism of retinoids and/or nitric oxide. There is some indirect evidence to support this hypothesis. Consider the case of Lesch-Nyhan syndrome (LNS), in which persistent and severe self-injury involving finger and lip biting is a prominent feature, occurring in 85% of affected males. LNS is a rare inherited disorder of purine metabolism caused by a deficiency of the enzyme hypoxanthine-guanine phosphoribosyltransferase (HPRT), produced by mutations in the HPRT gene located on the X chromosome. LNS affects about one in 380,000 live births and was first described by Lesch and Nyhan in 1964 (http://en.wikipedia.org/wiki/Lesch%E2%80%93Nyhan_syndrome).

The classical clinical phenotype caused by HPRT deficiency includes massive uric acid overproduction, motor dysfunction, cognitive disability, and self-injurious behavior [183]. Hyperuricemia is associated with severe gout and kidney problems. Subsequent studies showed that the degree of HPRT deficiency determines the severity of LNS, and only complete deficiency causes self-biting [184]. Signs of poor muscle control and moderate mental retardation appear in the first year of life, followed in the second year by self-mutilating behaviors, facial grimacing, involuntary writhing, and repetitive movements of the arms and legs. SIB can result in partial or total destruction of perioral tissues. Severe panic attacks and neophobia are common in later life. The mechanisms of the purine metabolic aberrations are well understood, but those causing the neurological abnormalities are unknown.

The first point in support of the retinoid model is that SIB associated with hyperactivity and stereotypical behaviors can be induced in rats and mice by interventions that affect striatal dopamine systems, which are influenced by retinoic acid. These interventions include prolonged high doses of caffeine and theophylline, brain lesions caused by the dopaminergic toxin 6-hydroxydopamine (6-OHDA) during early postnatal development, and administration of ±BayK 8644, an activator of voltage-regulated (L-type) calcium channels [185].

Second, HPRT deficiency is accompanied by aberrations in pathways implicated in neurogenesis and neurodegenerative disease, including the canonical Wnt/beta-catenin and the Alzheimer's disease/presenilin signaling pathways, that are associated with retinoids. The Wnt signaling pathway is a network of proteins involved in embryogenesis and cancer and also in normal physiological processes in adult animals. Wnt proteins are a class of secreted ligands that profoundly affect morphogenesis in all multicellular organisms. Both of these pathways are vital for the generation and function of dopaminergic neurons [186] and are linked to alterations in retinoid signaling. Exogenous pattern signals including Wnt (of which betacatenin is a central component) and retinoic acid interact closely in the differentiation of embryonic stem cells into a wide range of neural cell types [187]. There are also multiple links between RA signaling and Alzheimer's disease [188]. On the other hand, efforts to control self-injurious behaviors using dopamine receptor antagonists including pimozide, haloperidol, fluphenazine, and Risperdal have no significant influence on SIB and carry the risk of causing tardive dyskinesia syndromes [183].

Third, purine and vitamin A metabolism are closely related processes, and there is evidence that RA is an important product of purine metabolism. Xanthine oxidase (XO), the enzyme responsible for converting hypoxanthine to xanthine and xanthine to uric acid, can also oxidize retinol to its more toxic metabolite RA [189]. The XO inhibitor allopurinol also inhibits the synthesis of RA. Taibi et al. [190] reported that milk XO efficiently catalyzed the conversion of retinaldehyde to RA. The enzyme oxidized retinaldehyde in the presence of oxygen with or without NAD, the catalytic efficiency of the enzyme being higher in the latter condition. The synthesis of RA was strongly inhibited in a dose-dependent manner by allopurinol. Hence, it is possible that SIB associated with the genetic defect in HPRT could be due to the accumulation RA or associated toxic vitamin A compounds in brain such as retinyl esters, as well as uric acid. If the retinoid toxicity hypothesis is correct, the ratio of serum retinoic acid to retinol and percent retinyl esters as a fraction of total vitamin A should be significantly higher in patients with LNS than in controls. Although allopurinol affects retinoid metabolism, controls the overproduction of uric acid, and reduces the risk of nephrolithiasis, gouty arthritis, and tophi in LNS, it has no effect on the behavioral or neurologic symptoms of LNS [191]. It is possible that drug treatments that affect retinoid metabolism and expression (e.g., retinoic acid receptor antagonists) and/or increase nitrergic activity could be useful in the treatment of LS syndrome and other forms of SIB.

## 11. Further Implications for Research and Practice

Several strands of research have been marshaled in support of the proposed retinergic-nitrergic imbalance model of acute and chronic conditions in which cognitive impairments, hyperactivity, and aggression are prominent features. Subject to obtaining empirical support based on further animal and human studies, novel pharmaceutical and/or physical treatment strategies could be considered for correcting the postulated biochemical imbalance.

The hypothesis of behavioral inhibition by sensory feedback suggests that the effectiveness of active and passive forms of stimulation for treating learning disability associated with hyperactivity and aggression (e.g., physical exercise, sensory integration interventions, therapeutic massage, chiropractic methods, and acupuncture) may be mediated in part by reducing circulating retinoid and/or increasing nitric oxide concentrations. Consistent with the model, physical exercise is reported to lower circulating retinoid concentrations. One study involved 29 exercise sessions performed over a 7-week period by six healthy male subjects, each session consisting of jogging on a treadmill for 30 minutes. Subjects averaged 15.2 miles/week. A vitamin A fat-loading test was used specifically to label and follow postprandial lipoprotein levels using retinyl ester concentrations. The exercise conditioning program led to a significant 37% decrease in chylomicron retinyl palmitate levels [192]. In a more recent study, 29 healthy women performed 60 minutes of aerobic exercise three times weekly for 10 weeks at about 70% maximal exercise capacity. After the 10-week training program, insulin sensitivity was improved, and serum levels of RBP were significantly decreased [193]. It is not yet known if passive forms of stimulation such as acupuncture or massage affect retinoid and/or nitric oxide metabolism.

The reported success of physical methods, such as exercise and massage for treating ADHD and related conditions (e.g., [20]), suggests the need to compare and quantify the outcomes of such treatments with those of stimulants. In the case of passive therapies, studies are needed to determine the most effective methods and optimal parameters for delivering stimulation, such as duration and intensity. In the case both of exercise and applied sensory stimulation, studies are needed to determine the optimal conditions for achieving clinically relevant improvements in cognitive abilities, performance, and behavior. For persons with cognitive impairments and behavioral disorders, substitute forms of sensory stimulation are not usually available at the time they could be most useful, that is, in the classroom or during episodes of intense SSB. The model suggests that, for such individuals, the delivery of therapeutic sensory stimulation would be most effective at the onset of the behavior. To this end, portable stimulators could be developed and activated at will or as needed by the user, parent, or teacher.

In addition to short-term improvements in attention, scholastic performance, and classroom behavior, it may be possible to achieve lasting improvement in overall symptoms using therapeutic sensory stimulation. Just as therapeutic massage promotes the growth and wellbeing of preterm infants [89, 92], long-term sensory stimulation may prove similarly effective for enhancing growth and cognitive development in children with ADHD, ASD, and related disorders.

## 12. Conclusions

The theory has been proposed that cognitive impairments associated with hyperactive behavior and aggression are due to disturbances in the metabolism of endogenous retinoids (vitamin A and its congeners) and/or nitric oxide (NO), resulting in retinergic overexpression, causing chronic increases in stimulation-seeking behavior (SSB). Bodily stimulation so obtained or generated may be part of a negative feedback system in which SSB results in its own inhibition via the activation of nitrergic (NO) systems. In the case of individuals with cognitive and behavioral disorders, the retinergic system may be chronically overactivated and the opposing nitrergic system chronically underactivated. The intensity of sensory stimulation generated by the subject through SSB may thus be insufficient to activate the nitrergic “brake” over the retinergic system, so that SSB remains intense, persistent, and potentially harmful to the self or others.

The model suggests that cognitive abilities, scholastic performance, and behavior could be improved in the classroom setting and, perhaps more enduringly, by using intense but non-harmful forms of sensory stimulation to activate the nitrergic system directly and thereby reduce retinergic activation. Provision of alternative forms of sensory stimulation would be expected to substitute for that obtained in maladaptive or harmful ways by agitation, self-injurious behavior, and aggression, and thereby enhance cognitive functions and abilities. Consistent with the model, many reports and reviews indicate that physical exercise, therapeutic massage, and other forms of sensory stimulation are useful in the treatment of ADHD and related conditions. There is also an evidence that physical exercise can lower serum retinyl ester concentrations, as predicted by the model. New technology is needed, therefore, to help focus attention, enhance cognitive skills and abilities, and reduce unwanted SSB. While such methods may be effective for many children and adults with learning disorders, physical therapies may need to be combined with pharmaceutical approaches in more intractable cases in order to correct the hypothesized retinergic-nitrergic imbalance and achieve desired and enduring improvements in cognitive functioning and behavior. In conclusion, a comprehensive multidisciplinary research program is needed to test the retinergic-nitrergic model and to determine the most effective modes of delivery, assistive technologies, and optimal parameters of stimulation for reducing SSB, improving attention, cognitive skills, and scholastic performance, as well as future employment prospects for children with learning and behavior disorders.
